# Supplementary material for: Effects of Trace Metals and Municipal Wastewater on the Ephemeroptera, Plecoptera, and Trichoptera of a Stream Community
Source: Biology (Basel). 2022 Apr 24;11(5):648. doi: 10.3390/biology11050648 (PMC9137756; doi:10.3390/biology11050648)
Supplement: Supplementary file 1 [file biology-11-00648-s001.zip › biology-1695692-supplementary.pdf]

Effects of trace metals and municipal wastewater on the Ephemeroptera, Plecoptera and Trichoptera of a stream community

Marek Let*, Jan Černý, Petra Nováková, Filip Ložek and Martin Bláha*

Faculty of Fisheries and Protection of Waters, South Bohemian Research Center of Aquaculture and Biodiversity of Hydrocenoses, University of South Bohemia in České Budějovice, Vodňany, Czech Republic Zátiší 728/II 389 25 Vodňany Czech Republic; cernyj18@frov.jcu.cz (J.Č.); novakovapetra@frov.jcu.cz (P.N.); lozekf@frov.jcu.cz (F.L.)
* Correspondence: mlet@frov.jcu.cz (M.L.); blaha@frov.jcu.cz (M.B.)

1. Supplementary materials

1.1. Materials and methods

Grab samples of sediments and water for the analysis of metals, pesticides, pharmaceutical active compounds (PhACs), and total organic carbon were also taken at three auxiliary sites to improve our knowledge of contamination along the longitudinal profile of the studied river basin. Auxiliary site 1: the Litavka river immediately above its confluence with the Obecnický brook (N 49.7076664, E 13.9832436; third-order watercourse), Auxiliary site 2: the Obecnický brook immediately above its confluence with the Litavka river (N 49.7085058, E 13.9828894; fourth-order watercourse), Auxiliary site 3: the Litavka river immediately above its confluence with the Příbramský brook (N 49.7105836, E 14.0076639; fourth-order watercourse), and Auxiliary site 4: the Příbramský brook immediately above its confluence with the Litavka river (N 49.7104658, E 14.0099639; third-order watercourse).

All grab samples of sediments and water were stored during fieldwork in sealed polystyrene boxes filled with ice. Ten-mL samples for analysis of the PhACs, pesticides and dissolved metals were filtered through regenerated a cellulose filter (0.20-μm pores, HPST) immediately after sampling. Samples of sediments and water for the analysis of metals (cadmium, lead and zinc) and organic carbon were transported to an accredited water management laboratory in Prague (Povodí Vltavy, State Enterprise, Czech Republic) two days after sampling for analysis. Samples for analysing PhACs and pesticides were stored in a freezer (N3023-22, Liebherr) at –32 °C prior to analysis at the Faculty of Fisheries and Protection of Waters.

The analysis of the metals in the sediment samples involved lyophilisation, homogenisation, mineralisation by a microwave system, and inductively coupled plasma mass spectrometry (ICP-MS) performed according to standardised methods ČSN EN ISO 17294-1,2); measurement uncertainty was set at ±20–30 %. Thermal decomposition after total inorganic carbon removal was performed for the establishment of total organic carbon in the sediment samples according to standardised methods ČSN EN 19137 and ČSN ISO 10694; measurement uncertainty was set at ± 30 % for all samples. The water samples were analysed using ICP-MS according to the method ČSN EN ISO 17294-1,2; measurement uncertainty was set at ± 15–25 %, except for concentrations below limits of quantification (LOQs).

The chemical analyses of the water samples for detecting PhACs and pesticides was conducted using liquid chromatography tandem mass spectrometry (LC-MS/MS) with a TSQ Quantiva Triple-Stage Quadrupole Mass Spectrometer, Accela 1250, and Accela 600 LC pumps (Thermo Fisher Scientific, USA) and a HTS XT-CTC autosampler (CTC Analytics AG, Switzerland). The sample preparations, with details about the analytical methods used, have been described in previous publications [1-3]; Five mL of defrosted and filtered samples were spiked with internal standards and analysed. The results were processed and quantified using the software Trace Finder 3.1. (Thermo Fisher Scientific, USA). The LOQs are listed in Table S6.

The fish stocks were monitored on 20 May and 8 October using a petrol-powered electrofishing device (HONDA GX160 3600) at the sites shown in Table S9 . Eight point-like samplings (semicircles with a radius of around two meters) were performed at the chosen sites. Captured fish were identified, measured, and weighed using scales (Kern EMS). Site 2.2 was situated approximately 2000 m downstream from Site 2 (The Obecnický brook, fourth-order watercourse; from N 49.7107567, E 13.9657250 to N 49.7107947, E 13.9667039). Site 4 was divided into subsites 4.1 and 4.2. Site 4.1. corresponds to the channelled part of Site 2 (upstream section), while Site 4.2 corresponds to the non-channelled part of this same site (downstream section).

We also tested relationships between EPT richness, EPT family richness, EPT abundances, and relative EPT abundances (established for 19 samples from a total of 48 samples). Metal index calculated from water concentrations according to the following equation from Giddings et al. [4]:

$$\text{Metal index} = \sum_{i=1}^{n} (Xi/Ximax) * y$$

Where n = number of metals in the index, X_i = concentration of metal i at a site, X_{imax} = maximum concentration of the metal i observed at all sites. The generalized linear models (GLMs) with binomial, Poisson, or negative binomial distributions were applied according to the response data. The significance of an improvement by using generalized mixed-effect models (GLMMs) with sampling time as a variable with random effect were compared with GLMs by the likelihood ratio test. The quasi-likelihood estimation method was used in GLMs with Poisson or binomial distribution when overdispersion was detected.

1.2. Results

There was significant relatioship between EPT family richness and Metal index ($\chi^2_1 = 13.42$, $p < 0.001$). The relationships between EPT richness, EPT abundances, and relative EPT abundances and Metal index were not significant ($F_{1,46} = 2.08$, $p = 0.16$; $F_{1,46} = 0.02$, $p = 0.88$; $F_{1,17} = 0.05$, $p = 0.83$; respectively).

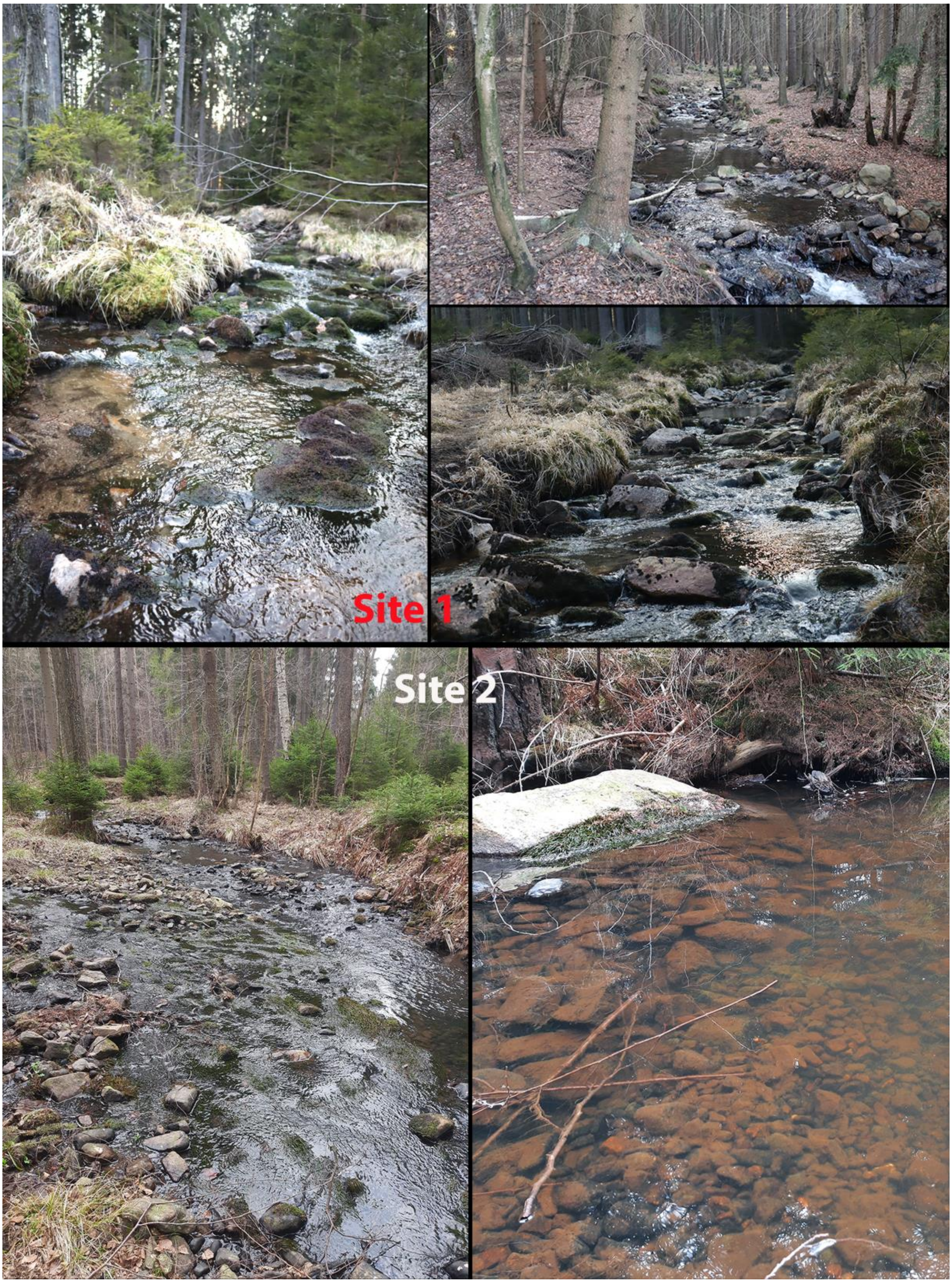

Figure S1. Photographic documentation of Site 1 (upper part) and Site 2 (down part). Pictures were taken in March 2022. The picture in the upper left corner shows the confluence of the Obecnický brook and a spring-fed nameless tributary. There were subnormal discharges at both sites.

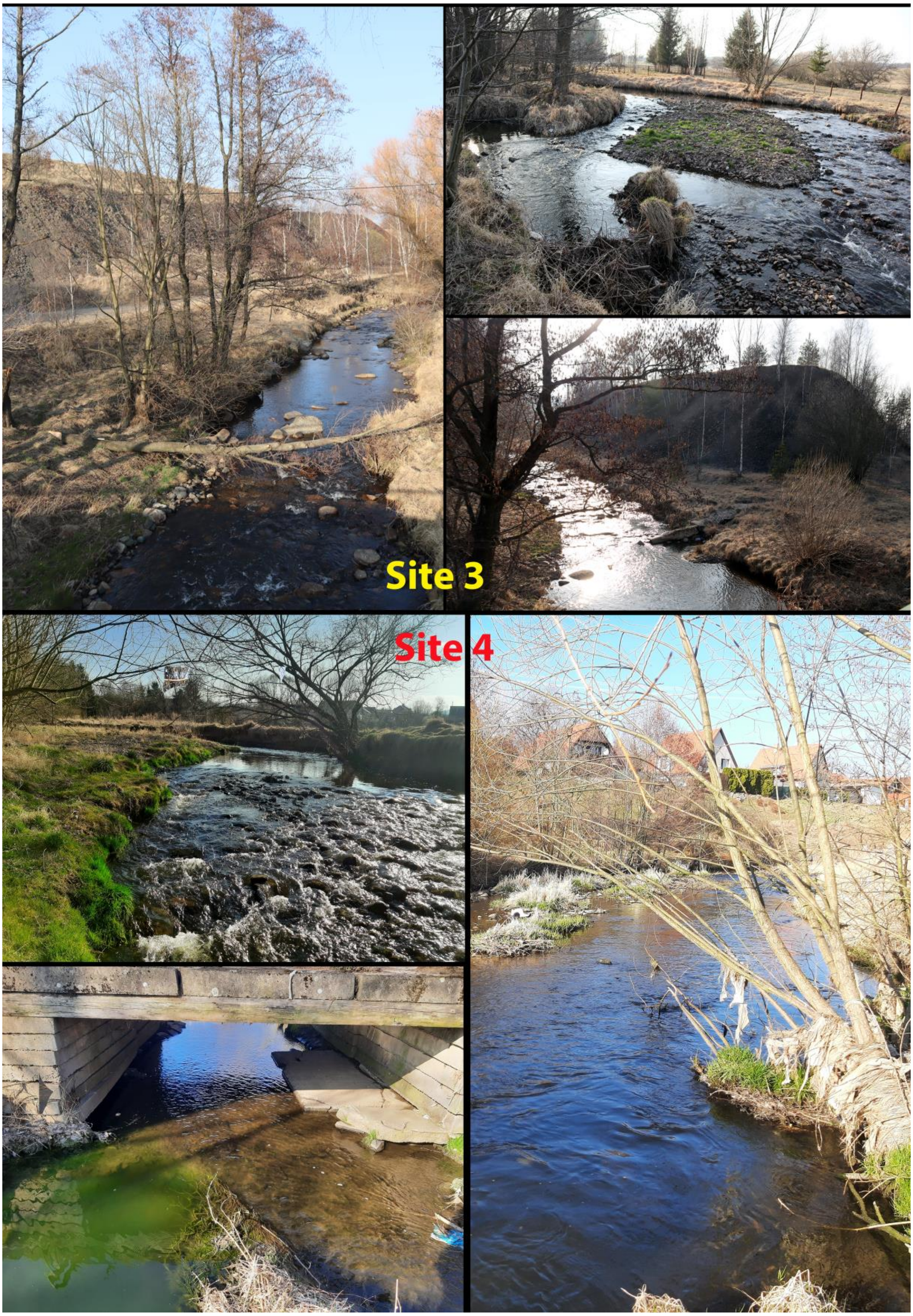

Figure S2. Photographic documentation of Site 3 (upper part) and Site 4 (down part). Pictures were taken in March 2022. The non-channelised part of the Site 3 is shown in the picture in upper right corner. Heaps of waste materials (mainly sodium slag) are visible in the pictures of the channelised part of Site 3. A periodically active effluent of 'poorly treated communal wastewaters' flowing into Auxiliary site 4 is shown in the picture in down left corner.

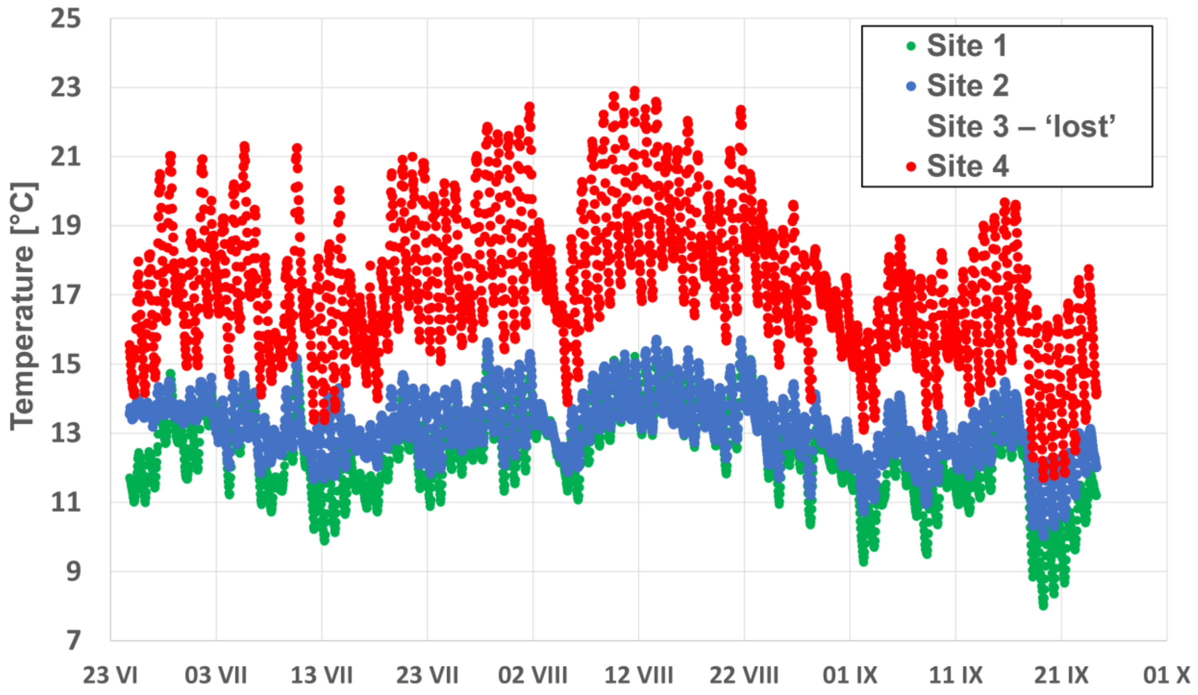

Figure S3. Temperature monitored by dataloggers (TFA) during the time interval between 24 June to 24 September 2020 at given sites. The datalogger placed in the Site 3 was lost.

Table S1. Physicochemical parameters of water measured at particular sites. Comparison between Sites 1–4 and Auxiliary sites 1–4 is not fully relevant because the times of measuring parameters at auxiliary sites do not fully correspond to the times of measuring parameters at Sites 1–4. Aux. site = Auxiliary site.

	**Site 1**	**Site 2**	**Aux. site 1**	**Aux. site 2**	**Site 3**	**Aux. site 3**	**Aux. site 4**	**Site 4**
	**n = 4**	**n = 4**	**n = 3**	**n = 3**	**n = 4**	**n = 3**	**n = 3**	**n = 4**
	**Mean ± SEM**	**Mean ± SEM**	**Mean ± SEM**	**Mean ± SEM**	**Mean ± SEM**	**Mean ± SEM**	**Mean ± SEM**	**Mean ± SEM**
**Temperature (°C)**	12.05 ± 0.34	13.33 ± 0.61	17.85 ± 2.73	15.24 ± 1.91	17.05 ± 1.11	15.76 ± 1.33	19.59 ± 1.37	16.80 ± 0.68
**pH**	7.09 ± 0.24	7.35 ± 0.17	8.22 ± 0.05	7.74 ± 0.02	7.45 ± 0.17	8.81 ± 0.69	7.44 ± 0.15	7.45 ± 0.09
**Oxygen concentration (mg·L^{-1})**	9.90 ± 0.10	9.02 ± 0.18	9.97 ± 0.40	9.11 ± 0.35	8.91 ± 0.16	9.62 ± 0.67	5.36 ± 0.78	7.61 ± 0.27
**Conductivity (μS·cm^{-1})**	71.40 ± 5.80	84.35 ± 5.98	392.33 ± 61.52	362.00 ± 48.42	390.60 ± 39.18	609.32 ± 76.97	838.67 ± 9.66	571.00 ± 56.74

Table S2. Parameters of discharge at particular sites continually measured by Povodí Vltavy, State Enterprise between February and September of 2020.

	**Site 1**	**Site 2**	**Site 3**	**Site 4***
**Average discharge ± SD (m^3·s^{-1})**	0.11 ± 0.14	0.08 ± 0.12	Not available	0.88 ± 0.87
**The lowest observed discharge (m^3·s^{-1})**	0.017	0.015	Not available	0.187
**The highest observed discharge (m^3·s^{-1})**	1.328	1.340	Not available	7.480

*** The hydrological station measuring discharge is situated approximately 5.4 km downstream the Site 4. Therefore, the given parameters can be slightly higher**

Table S3. Concentrations of metals and organic carbon in sediment samples and concentrations of metals in water samples. Samples were taken during 24 September 2020 in the particular sites. The sites are ordered from the most upstream site to the most downstream site. Aux. site = Auxiliary site.

	**Site 1**	**Site 2**	**Aux. site 1**	**Aux. site 2**	**Site 3**	**Aux. site 3**	**Aux. site 4**	**Site 4**
**Concentrations in sediment samples (mg·kg⁻¹ DW)**								
**Cadmium**	1.70	2.60	76.00	240.00	30.00	71.00	24.00	51.00
**Lead**	170	87	9100	140000	3700	5900	1100	2200
**Zinc**	180	170	11000	3400	3800	7400	3600	5200
**Total organic carbon**	27000	25000	39000	62000	19000	74000	69000	78000
**Concentrations in water samples (μg·L⁻¹)**								
**Cadmium**	0.26	0.23	3.50	3.10	7.90	7.20	1.30	3.80
**Cadmium dissolved**	< 0.05	< 0.05	2.40	0.69	6.50	4.30	0.11	0.50
**Lead**	5.80	9.80	22.00	690.00	64.00	100.00	38.00	53.00
**Lead dissolved**	< 0.50	3.20	3.50	8.60	14.00	7.90	1.00	1.70
**Zinc**	15	13	1000	120	1200	910	210	530
**Zinc dissolved**	< 5	<5	820	31	1000	640	48	180

Table S4. Concentrations of cadmium (Cd), lead (Pb) and zinc (Zn) in water samples estimated in water samples from Auxiliary site 4 (Aux. site 4) and Site 4. Data provided by the Czech Hydrometeorological Institute (Prague, Czech Republic) and Povodí Vltavy, State Enterprise (Prague, Czech Republic).

		**2018**			**2019**			**2020**		
		Cd (μg·L⁻¹)	Pb (μg·L⁻¹)	Zn (μg·L⁻¹)	Cd (μg·L⁻¹)	Pb (μg·L⁻¹)	Zn (μg·L⁻¹)	Cd (μg·L⁻¹)	Pb (μg·L⁻¹)	Zn (μg·L⁻¹)
**Aux. site 4**	Mean ± SEM	0.53 ± 0.09	22.43 ± 4.06	143.58 ± 13.21	0.87 ± 0.22	34.57 ± 7.83	146.75 ± 20.45	0.67 ± 0.10	24.82 ± 4.33	148.42 ± 19.37
	Min	0.13	3.60	53.00	0.17	6.10	41.00	0.23	7.30	81.00
	Max	1.10	54.00	220.00	3.20	92.00	260.00	1.30	53.00	340.00
**Site 4**	Mean ± SEM	3.20 ± 0.42	26.19 ± 5.18	430.00 ± 21.21	3.89 ± 0.46	90.61 ± 62.01	610.00 ± 67.27	3.40 ± 0.21	52.67 ± 10.75	484.17 ± 22.53
	Min	1.80	7.30	320.00	1.80	8.30	290.00	2.40	14.00	390.00
	Max	7.60	71.00	580.00	8.30	800.00	1200.00	4.80	130.00	640.00

Table S5. Pesticides and pharmaceutical active compounds (PhACs) in water samples taken in August 2020 in the particular sites. Only the compounds below the limits of quantifications are involved. The sites are ordered from the most upstream site to the most downstream site. Max = the concentration of the compound with the highest concentration. Aux. site = Auxiliary site.

	**Site 1**	**Site 2**	**Aux. site 1**	**Aux. site 2**	**Site 3**	**Aux. site 3**	**Aux. site 4**	**Site 4**
	n = 1	n = 1	n = 1	n = 1	n = 1	n = 1	n = 1	n = 4 Mean ± SEM
**Sum pesticides (ng·L⁻¹)**	132.00	70.32	695.40	1096.10	462.90	1304.00	1692.62	877.87 ± 125.71
**N pesticides**	8	11	13	22	21	21	37	29.25 ± 0.83
**Max pesticides (ng·L⁻¹)**	48	13	230	320	130	440	650	207.50 ± 37.00
**Sum PhACs (ng·L⁻¹)**	15.17	13.40	1781.47	230.07	874.58	471.76	10,036.78	4636.73 ± 254.79
**N PhACs**	6	4	31	18	28	20	48	45.25 ± 1.38
**Max PhACs (ng·L⁻¹)**	7.4	8.7	610.0	59.0	310.0	130.0	3100.0	1275.00 ± 47.87

Table S6. List of compounds analysed in water samples taken during 11th August 2020 with particular limits of quantification (LOQs).

Pesticides and their metabolites			Pharmaceutical active compounds (prescription pharmaceuticals and illicit drugs)		
**Compound**	**Min LOQ ($ng \cdot L^{-1}$)**	**Max LOQ ($ng \cdot L^{-1}$)**	**Compound**	**Min LOQ ($ng \cdot L^{-1}$)**	**Max LOQ ($ng \cdot L^{-1}$)**
1-(3.4-Dichlorophenyl) urea	5.9	20.0	Alfuzosin	0.26	0.56
1H-benzotriazol	1.8	5.9	Atenolol	0.60	3.90
1H-benzotriazol (5/4)-methyl	0.64	2.20	Atorvastatin	0.74	1.70
1H-benzotriazol 1-methyl	3.3	11.0	Azithromycin	0.73	1.50
2.4.5-trichlorophenoxyacetic acid	5	16	Bezafibrate	0.85	2.00
2.4-D	4.4	14.0	Biperiden	0.43	0.99
2.4-Dichlorphenoxypropionic acid	3.9	12.0	Bisoprolol	0.73	1.50
3-chloro-4-methylaniline	1.1	5.8	Cetirizine	0.96	2.20
4-Isopropylaniline	2.4	7.9	Cilazapril	0.65	1.50
Acetochlor	2.2	11.0	Clarithromycin	2.8	7.2
Acetochlor ESA	2.8	9.0	Clemastine	0.21	0.48
Acetochlor OA	7.5	24.0	Clindamycin sulfoxide	0.54	1.10
Alachlor	2.1	10.0	Clindamycine	0.98	2.00
Alachlor ESA	47	150	Diclofenac	4.4	10.0
AlachlorOA	6	19	Dicycloverine	0.27	0.62
Ametryn	0.97	3.30	Diltiazem	0.69	1.60
Anthranilic acid isopropylamide	1.2	4.1	Diphenhydramine	0.34	0.78
Atraton	0.86	2.90	Disopyramide	0.48	1.00
Atrazine	0.75	3.40	Erythromycin	0.81	1.60
Atrazine 2-hydroxy	0.62	3.30	Fenofibrate	1.4	3.2
Atrazine desethyl	0.41	1.40	Fexofenadine	0.19	0.43
Atrazine desethyl-2-hydroxy	2.8	13.0	Glibenclamide	0.9	2.1
Atrazine desisopropyl	3.4	12.0	Glimepiride	0.84	1.90
Azoxystrobin	1.0	4.9	Iopromide	7.5	49.0
Bensulfuron methyl	0.34	1.70	Irbesartan	0.73	1.70
Bentazone	0.87	2.80	Loperamide	0.095	0.220
Carbendazim	0.89	4.10	Meclozine	0.57	1.30
Carbofuran-3-hydroxy	1.2	3.9	Memantine	0.32	0.70
Chlorantraniliprole	1.7	8.5	Metoprolol	0.6	1.2
Chloridazon	7	24	Metoprolol acid	0.64	4.20
Chloridazon methyl desphenyl	2.4	11.0	Miconazole	0.18	0.42
Chlorotoluron	1.3	4.5	N1 Acetylsufamethaxazole	5.1	14.0
Chlorotoluron desmethyl	4.7	16.0	N4 Acetylsufamethaxazole	7.2	19.0
Clomazone	0.49	2.40	Orphenadrine	0.34	0.78
Cyanazine	1.3	4.3	Oseltamivir carboxylate	1.4	3.9
Cyproconazole	0.34	1.70	Pizotifen	0.33	0.77
DEET	20	20	Propranolol	0.49	0.99
Desmetryn	0.94	3.20	Ropinirole	0.62	1.30
Diazinon	0.7	3.5	Rosuvastatin	0.76	1.80
Dimethachlor	0.3	1.5	Roxythromycin	0.35	0.90
Dimethachlor ESA	1.6	8.5	Sotalol	1.6	10.0
Dimethachlor OA	8.2	28.0	Sulfadiazine	5.2	14.0
Dimethenamid ESA	7.3	25.0	Sulfamerazine	1.4	3.8
Dimethenamid OA	7.9	27.0	Sulfamethazine	0.76	2.00
Dimethoate	4	14	Sulfamethizole	0.84	2.20
Dimethomorph	1.0	4.9	Sulfamethoxazole	1.8	4.7
Diuron	5.1	17.0	Sulfapyridine	1.5	4.0
Diuron desmethyl	3.5	12.0	Tamoxifen	0.13	0.30
Epoxiconazole	0.65	3.20	Telmisartan	2.1	4.8
Fenuron	0.78	2.60	Terbinafine	0.33	0.76
Florasulam	2.2	7.5	Terbutaline	0.64	1.50

Fluazifop-p	0.4	2.0	Theophylline	2.5	16.0
Flusilazole	0.54	2.70	Theophylline neg	4.8	32.0
Foramsulfuron	1.7	5.8	Triamterene	0.51	3.30
Hexazinone	1.3	4.4	Trimethoprim	1.0	2.7
Imazamethabenz methyl	0.91	3.10	Valsartan	1.9	4.4
Imazamox	0.58	2.00	Verapamil	0.096	0.220
Imidacloprid	3.6	12.0	2-oxo-3-hydroxy-LSD	0.34	0.90
Ioxynil	1.1	3.6	6-acetylmophine	0.51	1.30
Isoproturon	2.1	7.0	Alprazolam	0.5	1.1
Isoproturon didemethyl	1.0	3.5	Amitryptyline	0.30	0.57
Isoproturon monodemethyl	1.3	4.4	Amphetamine	9.7	18.0
Lenacil	1.8	6.1	Benzoylecgonine	0.35	0.99
Linuron	3.7	18.0	Caffeine	1.8	4.3
Malathion	12	57.0	Cannabinol	5.7	12.0
MCPA	5	16	Carbamazepine	0.21	0.48
MCPP	3.8	12.0	Catinone	4.5	8.2
Metalaxyl	1.8	5.9	Citalopram	0.43	0.97
Metazachlor	0.76	3.70	Clomipramine	0.30	0.67
Metazachlor ESA	27	94	Clonazepam	1.9	3.0
Metazachlor OA	4.2	14	Cocaine	0.27	1.40
Metconazole	0.34	1.70	Codeine	0.44	2.90
Methabenzthiazuron	5.9	20.0	Dihydro CBZ	0.9	2.0
Methoxyfenozide	1.0	5.1	Donepezil	0.19	0.41
Metobromuron	4.8	16.0	Epoxy CBZ	1.1	2.5
Metolachlor	1.1	5.6	Haloperidol	0.42	0.94
Metolachlor ESA	2.9	9.8	Ketamine	0.43	1.20
Metolachlor OA	1.8	6.2	Lamotrigine	4.0	8.7
Metoxuron	1.1	3.7	Maprotiline	0.15	0.28
Metribuzin	11	38	MDA	13	27
Metribuzin desamino	6.5	22.0	MDEA	0.29	0.82
Metsulfuron methyl	1.2	4.0	MDMA	0.28	0.76
Monolinuron	7.3	25.0	Mephedrone	0.17	0.47
N-chloroacetyl-2.6-diethylaniline	3.8	13.0	Metamphetamine	0.42	1.10
Pirimicarb	0.69	3.60	Methadone	0.32	0.60
Pirimiphos ethyl	0.52	2.60	Mianserin	0.22	0.50
Pirimiphos methyl	0.53	2.60	Mirtazapine	0.66	1.40
Prometryn	1.2	4.0	Morphine	4.1	7.4
Propachlor	0.47	2.30	N-Desmethylcitalopram	0.69	1.60
Propazine	0.7	3.4	Norketamine	0.4	1.1
Propazine 2-hydroxy	0.85	2.90	Norsertraline	0.62	1.40
Propiconazole	0.57	2.80	O-Desmethylvenlafaxine	0.68	1.30
Pyrimethanil	0.64	2.10	Oxazepam	0.26	0.40
Sebuthylazine	4.2	21.0	Oxcarbazepine	0.6	1.3
Simazine	0.67	2.30	Oxycodone	0.92	2.00
Simazine hydroxy	1.0	3.5	Sertraline	1.4	3.1
Tebuconazole	2.1	11.0	THC-COOH	6.5	14.0
Terbuthylazine	0.53	2.60	Tramadol	0.43	0.92
Terbuthylazine desethyl	0.75	2.50	trans-dihydro-dihydroxy CBZ	3.5	7.9
Terbuthylazine desethyl-2-hydroxy	1.0	3.5	Trazodone	0.36	0.81
Terbuthylazine hydroxy	0.64	2.20	Venlafaxine	0.40	0.73
Terbutryn	1.1	3.8	Vortioxetine	0.31	0.70
Thiamethoxam	3.3	11			
Triadimenol	3	15			
Triallat	16	80			
Triticonazole	0.51	2.50			
Warfarin	1.5	7.4			

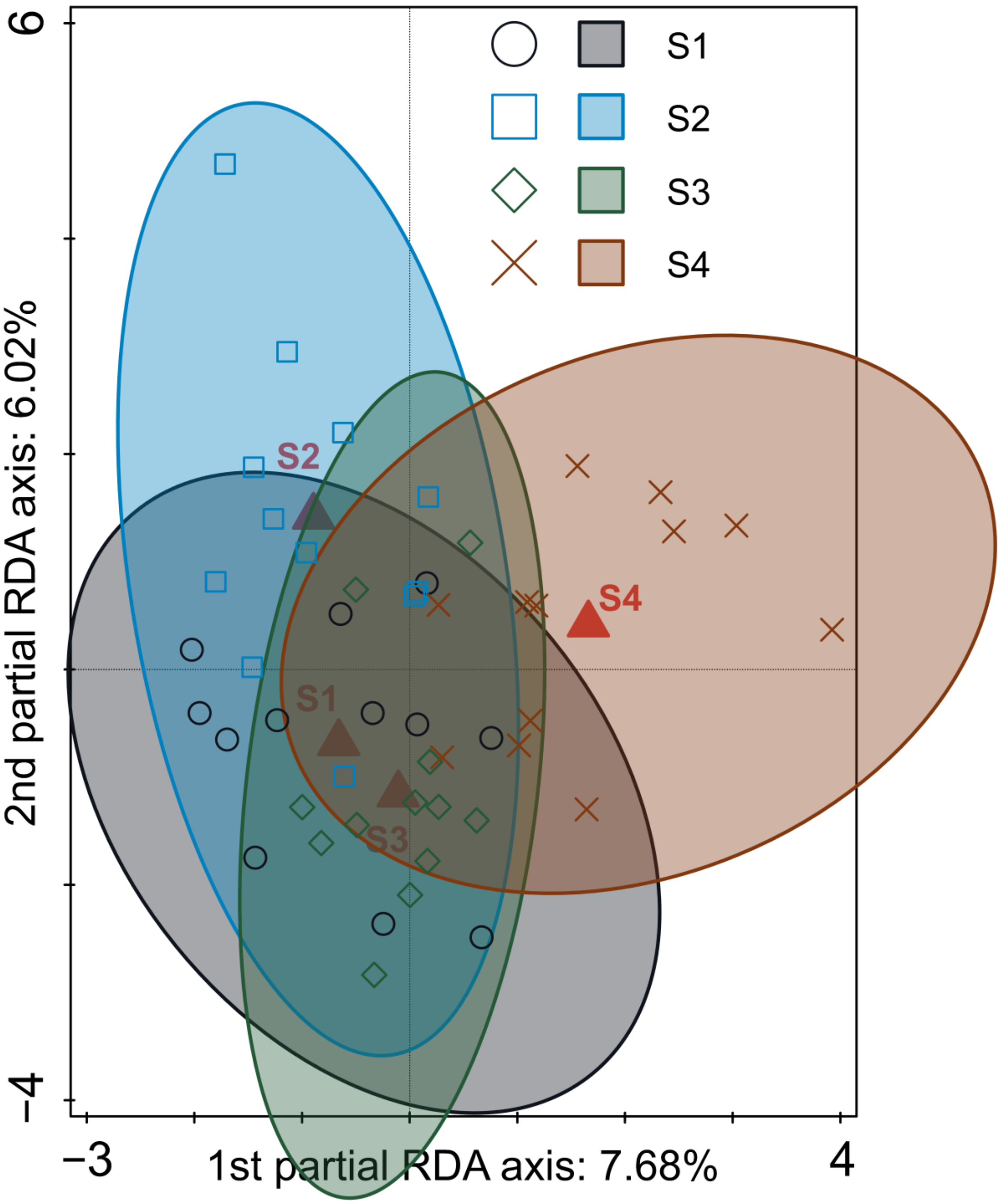

Figure S4. Classified sample diagram (caseR scores) resulted from choriotopic compositions. Partial Redundancy analysis (RDA) was used. Ordination axes were constrained by the factor 'Site' which accounted for 16.80% of the explained variation. Test on first axis: pseudo-*F* = 1.2, *p* = 0.085, test on all axis: pseudo-*F* = 2.8, *p* = 0.003. Conditional effects (from the greatest to the lowest size): 'Site 4 (S4) ': explained variation = 7.5%, pseudo-*F* = 3.8, *p* (adj.) = 0.025; 'Site 2 (S2)': explained variation = 6.0%, pseudo-*F* = 2.9, *p* (adj.) = 0.049 and 'Site 1 (S1)': explained variation = 3.4%, pseudo-*F* = 1.7, *p* (adj.) = 0.216. S3 = Site 3.

Table S7. List of EPT taxa detected at the given sites and times and their semi-quantitative abundances. V = 11 May, VI = 24 June, VIII = 11 August, and IX = 24 September. Explanations for semi-quantitative counts: '**X**' represents the range of 1–9 individuals, '**XX**' represents the range of 10–99 individuals, '**XXX**' represents the range of 100–999 individuals; empty cells represent no occurrence.

	**Site 1**				**Site 2**				**Site 3**				**Site 4**			
**Taxon**	**V**	**VI**	**VIII**	**IX**	**V**	**VI**	**VIII**	**IX**	**V**	**VI**	**VIII**	**IX**	**V**	**VI**	**VIII**	**IX**
**EPHEMEROPTERA**	XXX	XX	XX	XXX	XXX	XXX	XX	XX	XXX	XXX	XX	XXX	X	XXX	XXX	XX
**Baetidae**	X	X	XX	X	XX	XX	X	X	XXX	XXX	XX	XXX	X	XXX	XXX	XX
Baetis fuscatus (Linnaeus, 1761)		X	X			X			XXX	XX	XX	XX	X	XXX	XX	X
Baetis rhodani (Pictet, 1845)	X		X	X	XX	XX	X	X	X	XX		XX		XX	XX	X
Baetis* aff. *scambus (*fuscatus* gr.) (Eaton, 187)			X						XX	XX	XX	XX	X	X		
Baetis vernus (Curtis, 1834)	X		X	X		XX		X		X				XX	XX	X
Centroptilum luteolum (Müller, 1776)											X					
**Ephemerellidae**		XX	XX			XX									X	
Serratella ignita (Poda, 1761)		XX	XX			XX									X	
**Heptageniidae**	XX	X		XX	XX	X		XX								
Ecdyonurus torrentis (Kimmins, 1942)	X			X	X	X		XX								
Rhithrogena* cf. *puytoraci (Sowa & Degrange, 1987)		X														
Rhithrogena semicolorata (Curtis, 1834)	X	X		XX	XX			XX								
**Leptophlebiidae**	XXX	XX	XX	XXX	XX	XXX	XX	XX	X							
Habrophlebia lauta (Eaton, 1884)	XXX	XX	XX	XXX	XX	XXX	XX	XX	X							
Paraleptophlebia submarginata (Stephens, 1835)	X				X	X		X								
**PLECOPTERA**	XXX	XXX	XXX	XXX	XX	XXX	XXX	XXX	X	XX	XXX	XX		X	XX	
**Chloroperlidae**				X	X											
Siphonoperla torrentium (Pictet, 1841)				X	X											
**Leuctridae**	XX	XX	XX	XXX	XX	XXX	XXX	XX	X	XX	XXX	XX		X	XX	

	Site 1				Site 2				Site 3				Site 4			
**Taxon**	**V**	**VI**	**VIII**	**IX**	**V**	**VI**	**VIII**	**IX**	**V**	**VI**	**VIII**	**IX**	**V**	**VI**	**VIII**	**IX**
Leuctra albida (Kempny, 1899)	XX	X	X	XX	XX	XX	XX	X	X	XX	XX	X				
Leuctra* cf. *armata (Kempny, 1899)	X	XX														
Leuctra* cf. *autumnalis (Aubert, 1948)		X						X								
Leuctra braueri (Kempny, 1898)		X														
Leuctra fusca (Linnaeus, 1758)		X	X	XX	X	XXX	XXX	X	X	X	XX	XX		X	XX	
Leuctra geniculata (Stephens, 1836)									X	X	XX					
Leuctra inermis (Kempny, 1899)			X													
Leuctra* cf. *leptogaster (Aubert, 1949)		XX	X	X	X			XX								
Leuctra major (Brinck, 1949)				X												
Leuctra nigra (Olivier, 1811)	XX	X	X	X												
Leuctra* cf. *pseudocingulata (Mendl, 1968)		X	X	XX				X								
Leuctra* aff. *pseudosignifera (Aubert, 1954)								X								
***Leuctra* sp.** (Stephens, 1836) indet.	X	X	XX	XX		X		XX		X						
**Nemouridae**	XXX	XXX	XXX	XXX	XX	XX	XX	XX								
Amphinemura borealis (Morton, 1894)	X		X	XXX	X											
Amphinemura standfussi (Ris, 1902)			X		X											
Amphinemura sulcicollis (Stephens, 1836)	XX	XX		X	X	X										
Amphinemura sulcicollis/triangularis (Stephens, 1836/Ris, 1902)	X		X		X											
***Amphinemura* sp.** (Ris, 1902) indet.	X	X			X			X								
Nemoura avicularis (Morton, 1894)				X				X								

	Site 1				Site 2				Site 3				Site 4			
**Taxon**	**V**	**VI**	**VIII**	**IX**	**V**	**VI**	**VIII**	**IX**	**V**	**VI**	**VIII**	**IX**	**V**	**VI**	**VIII**	**IX**
Nemoura* aff. *cambrica (Stephens, 1836) – early instar nymphs								X								
Nemoura cinerea (Retzius, 1783)					X			X								
Nemoura* cf. *uncinata (Despax, 1934) – early instar nymphs			XX	XX			XX	XX								
***Nemoura* sp.** (Latreille, 1796) indet. – early instar nymphs			X				X									
Protonemura* cf. *aestiva (Kis, 1965)		X														
Protonemura hrabei (Raušer, 1956)		XX	XXX	XX		XX										
Protonemura intricata (Ris, 192)	XX	XX			X											
Protonemura* cf. *lateralis (Pictet, 1836)	XX	XX	X													
Protonemura meyeri/nitida (Pictet, 1841/ Pictet, 1836)			XX	XX				X								
Protonemura nitida (Pictet, 1836)		XX		X												
**Perlodidae**		X		XX												
Perlodes* aff. *microcephalus (Pictet, 1833) – early instar nymphs				X												
Diura bicaudata (Linnaeus, 1758). – early instar nymphs		X		X												
**TRICHOPTERA**	XX	XXX	XX	XX	XX	XXX	XX	XX	XXX	XXX	XXX	XXX	XX	XX	XXX	XXX
**Goeridae**		X	X	X			X				X					X
Goera pilosa (Fabricius, 1775)																X
Lithax niger (Hagen, 1859)		X	X	X												
**Goeridae gen. sp.** inc. (*Lithax niger*, Hagen, 1859 / *Silo nigricornis*, Pictet, 1834) – early instar larvae							X				X					
**Hydropsychidae**	X	X	X	X	X	XX	X	X	X	X	X	XXX	X	XX	XXX	XXX

	Site 1				Site 2				Site 3				Site 4			
**Taxon**	**V**	**VI**	**VIII**	**IX**	**V**	**VI**	**VIII**	**IX**	**V**	**VI**	**VIII**	**IX**	**V**	**VI**	**VIII**	**IX**
Hydropsyche angustipennis (Curtis, 1834)						X				X		X	X	X	XX	XX
Hydropsyche fulvipes (Curtis, 1834)						X										
Hydropsyche* aff. *guttata (Pictet, 1834) – early instar larvae											X	X				
Hydropsyche incognita (Pitsch, 1993)															XX	XX
Hydropsyche* aff. *incognita/pellucidula (Pitsch, 1993/Curtis, 1834) – early instar larvae								X							X	X
Hydropsyche instabilis (Curtis, 1834)					X	X										
Hydropsyche pellucidula (Curtis, 1834)											X	X			XX	XX
Hydropsyche saxonica (McLachlan, 1884)	X	X	X	X	X	XX		X								
Hydropsyche saxonica/fulvipes (McLachlan, 1884/Curtis, 1834) – early instar larvae		X	X			X										
Hydropsyche siltalai (Döhler, 1964)						X			X	X	X	XXX	X	X	XX	XXX
***Hydropsyche* sp.** indet. (Pictet, 1834) – early instar larvae							X	X				X			X	
**Hydroptilidae**													X			
***Hydroptila* spp**. (Dalman, 1819)													X			
**Leptoceridae**								X	XX	X		X			X	X
Athripsodes bilineatus (Linnaeus, 1758)								X	XX	X		X				X
Mystacides azurea (Linnaeus, 1761)												X			X	
															X	
**Limnephilidae**	XX	XX	X		XX	X	XX	X	XX	XXX	XX	X	X	X	X	

	Site 1				Site 2				Site 3				Site 4			
**Taxon**	**V**	**VI**	**VIII**	**IX**	**V**	**VI**	**VIII**	**IX**	**V**	**VI**	**VIII**	**IX**	**V**	**VI**	**VIII**	**IX**
Annitella obscurata (McLachlan, 1876)	X				X											
Annitella thuringica (Ulmer, 199)	X				X				X							
Drusus annulatus (Stephens, 1837)									X		XX	X				
Chaetopterygopsis maclachlani (Stein, 1874)	X	X														
Chaetopteryx major (McLachlan, 1876)		X								X					X	
Chaetopteryx villosa (Fabricius, 1798) – "dark-coloured"					X		X									
Chaetopteryx* cf. *villosa (Fabricius, 1798) – "light-coloured"					X		X		XX	XX	XX	X				
Halesus* cf. *digitatus (Schrank, 1781)											X					
Halesus* cf. *tesselatus (Rambur, 1842)									XX	XX	XX	X	X	X	X	
Halesus radiatus (Curtis, 1834)		X								X	X					
Hydatophylax infumatus (McLachlan, 1865) – early instar larvae			X													
Potamophylax* cf. *cingulatus (Stephens, 1837)					X				X	X	X					
Potamophylax* cf. *latipennis (Curtis, 1834)	X							X	X		X					
Potamophylax* cf. *luctuosus (Piller & Mitterpacher, 1783)		X									X					
Potamophylax rotundipennis (Brauer, 1857)		X	X			X				X			X			
Potamophylax* aff. *rotundipennis (Brauer, 1857)									X	XXX	X					
**Limnephilidae gen sp.** inc. (*Chaetopteryx villosa*/*Anitella obscurata*) – early instar larvae													X			

	Site 1				Site 2				Site 3				Site 4			
**Taxon**	**V**	**VI**	**VIII**	**IX**	**V**	**VI**	**VIII**	**IX**	**V**	**VI**	**VIII**	**IX**	**V**	**VI**	**VIII**	**IX**
**Limnephilidae gen sp.** inc. (*Micropterna* sp./*Potamophylax rotundipennis*) – early instar larvae													X			
**Limnephilidae gen. sp.** – early instar larvae	X	X										X				
**Odontoceridae**	X	XX	X	XX	X	X		X								
Odontocerum albicorne (Scopoli, 1763)	X	XX	X	XX	X	X		X								
**Philopotamidae**		XX	XX													
Philopotamus ludificatus (McLachlan, 1878)		XX	XX													
Philopotamus ludificatus/variegatus (McLachlan, 1878/Scopoli, 1763) – early instar larvae		X														
**Polycentropodidae**	X	X			X	XX	X	X	XX	XX	XX	XX	X	X	XX	XX
Cyrnus trimaculatus (Curtis, 1834)										X				X		XX
Neureclipsis bimaculata (Linnaeus, 1758)		X														
Plectrocnemia conspersa (Curtis, 1834)	X	X							X							
Plectrocnemia* cf. *geniculata (McLachlan, 1871)	X															
***Plectrocnemia* sp.** (Stephens, 1836) – early instar larvae					X				X		X	X			X	X
Polycentropus* cf. *excisus (Klapálek, 1894)					X	X			X	X	X	X				
Polycentropus flavomaculatus (Pictet, 1834)						XX	X	X	XX	XX	X	XX	X		XX	XX

	Site 1				Site 2				Site 3				Site 4			
**Taxon**	**V**	**VI**	**VIII**	**IX**	**V**	**VI**	**VIII**	**IX**	**V**	**VI**	**VIII**	**IX**	**V**	**VI**	**VIII**	**IX**
**Polycentropodidae gen. sp.** inc. (*Neureclipsis bimaculata*, Linnaeus, 1758) – early instar larvae												X			X	
**Polycentropodidae gen. sp.** inc. (*Neureclipsis bimaculata*, Linnaeus, 1758 / *Plectrocnemia* sp., Stephens, 1836)						X										
**Polycentropodidae gen. sp.** inc. (*Plectrocnemia* sp., Stephens, 1836) – early instar larvae										X	X	X				
**Polycentropodidae gen. sp.** inc. (*Polycentropus* sp., McLachlan, 1878) – early instar larvae					X	X										
**Psychomyiidae**															XX	XX
Psychomyia pusilla (Fabricius, 1781)															XX	XX
**Rhyacophilidae**	X	XX	XX	X	X	XX	X	X	X	X	XX	XX		X	XX	XX
Rhyacophila* cf. *aurata (Brauer, 1857)			X	X					X	X	XX	X			XX	XX
Rhyacophila* aff. *dorsalis (*nubila* gr.) (Curtis, 1834)			X													
Rhyacophila* cf. *fasciata (Hagen, 1859)															X	
Rhyacophila* aff. *nubila (Zetterstedt, 184)		X	X	X	X	X		X								
***Rhyacophila nubila* gr.** (Zetterstedt, 184)		X	X			X		X	X			X			XX	X
Rhyacophila* cf. *obliterata (McLachlan, 1863)						X										
Rhyacophila* cf. *polonica/praemorsa (McLachlan, 1879/ McLachlan, 1879)		X														
***Rhyacophila* sensu stricto** (Pictet, 1834) – early instar larvae		X	X		X	XX	X		X		X			X	X	

Taxon	Site 1				Site 2				Site 3				Site 4			
	**V**	**VI**	**VIII**	**IX**	**V**	**VI**	**VIII**	**IX**	**V**	**VI**	**VIII**	**IX**	**V**	**VI**	**VIII**	**IX**
***Rhyacophila* sp.** inc. (Pictet, 1834) – early instar larvae	X	X	X	X	X	X	X		X			X		X	X	X
***Rhyacophila* sp.** inc. (Pictet, 1834) – pupae		X		X								X		X		X
**Sericostomatidae**	X	XX	X	X	X	XX	X	X								
Sericostoma flavicorne/personatum (Schneider, 1845/Kirby & Spence, 1826)	X	XX	X	X	X	XX	X	X								
**Sericostomatidae gen. sp.** inc. (*Notidobia ciliaris* ?) (Linnaeus, 1761) – early instar larvae								X								
**Trichoptera fam. gen. sp.** indet. (Agapetinae/Goeridae ?) – early instar larvae		X														
**Trichoptera fam. gen. sp.** indet. (Brachycentridae?) – early instar larvae	X															

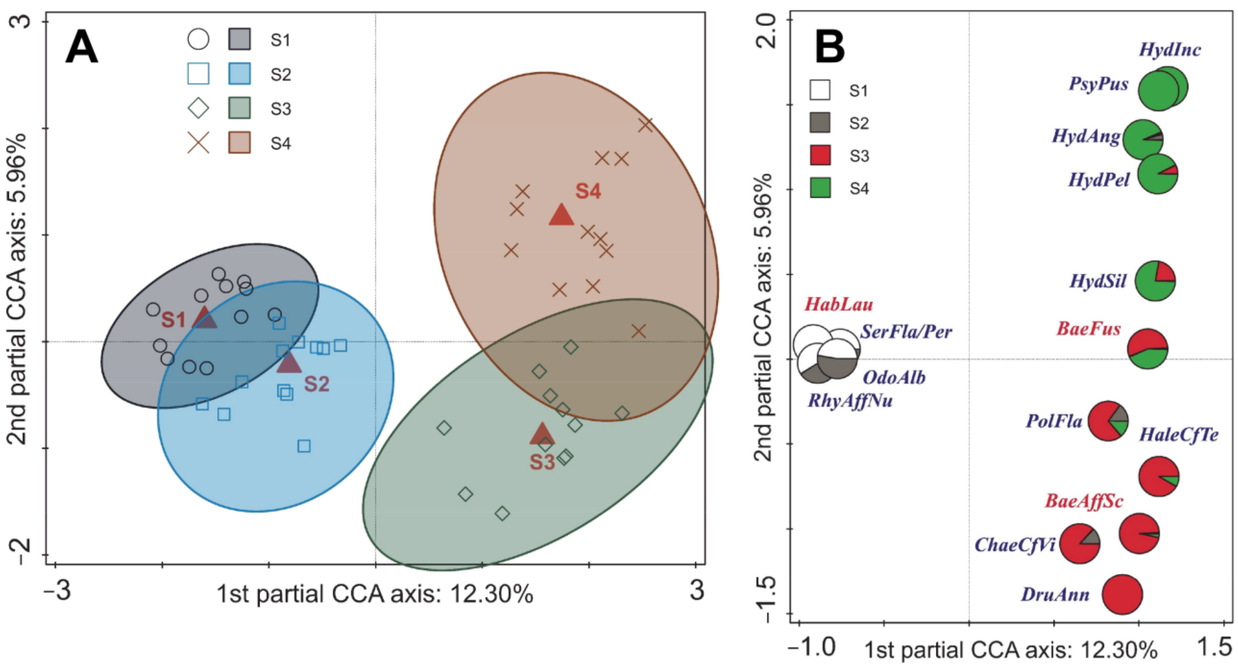

Figure S5. (A) Classified sample diagram (caseR scores) resulted from species compositions and (**B**) Species pie chart: the first 15 best fitting species are labelled (Ephemeroptera, Plecoptera and Trichoptera labaled in red, green and blue, respectively). Partial Canonical correspondence analysis (CCA) was used. Ordination axes were constrained by the factor 'Site' which accounted for 22.71% of the explained variation. Test on first axis: pseudo-F = 1.9, p = 0.001; test on all axes: pseudo-F = 4.0, p = 0.001. Conditional effects (from the greatest to the lowest explained variation): 'Site 1 (S1)': explained variation = 8.8%, pseudo-F = 4.1, p (adj.) = 0.002; 'Site 2 (S2)': explained variation = 8.0%, pseudo-F = 4.0, p (adj.) = 0.002 and 'Site 3 (S3)': explained variation = 5.9%, pseudo-F = 3.1, p (adj.) = 0.002. Abbreviations: *BaeAffSc* = *Baetis* aff. *scambus*, *BaeFus* = *B. fuscatus*, *ChaeCfVi* = *Chaetopteryx cf. villosa*, *DruAnn* = *Drusus annulatus*, *HabLau* = *Habrophlebia lauta*, *HaleCfTe* = *Halesus* cf. *tesselatus*, *HydAng* = *Hydropsyche angustipennis*, *HydInc* = *H. incognita*, *HydPel* = *H. pellucidula*, *HydSil* = *H. siltalai*, *OdoAlb* = *Odontocerum albicorne*, *PsyPus* = *Psychomyia pusilla*, *RhyAffNu* = *Rhyacophila* aff. *nubila*, *SerFla/Per* = *Sericostoma flavicorne/personatum*, S4 = Site 4.

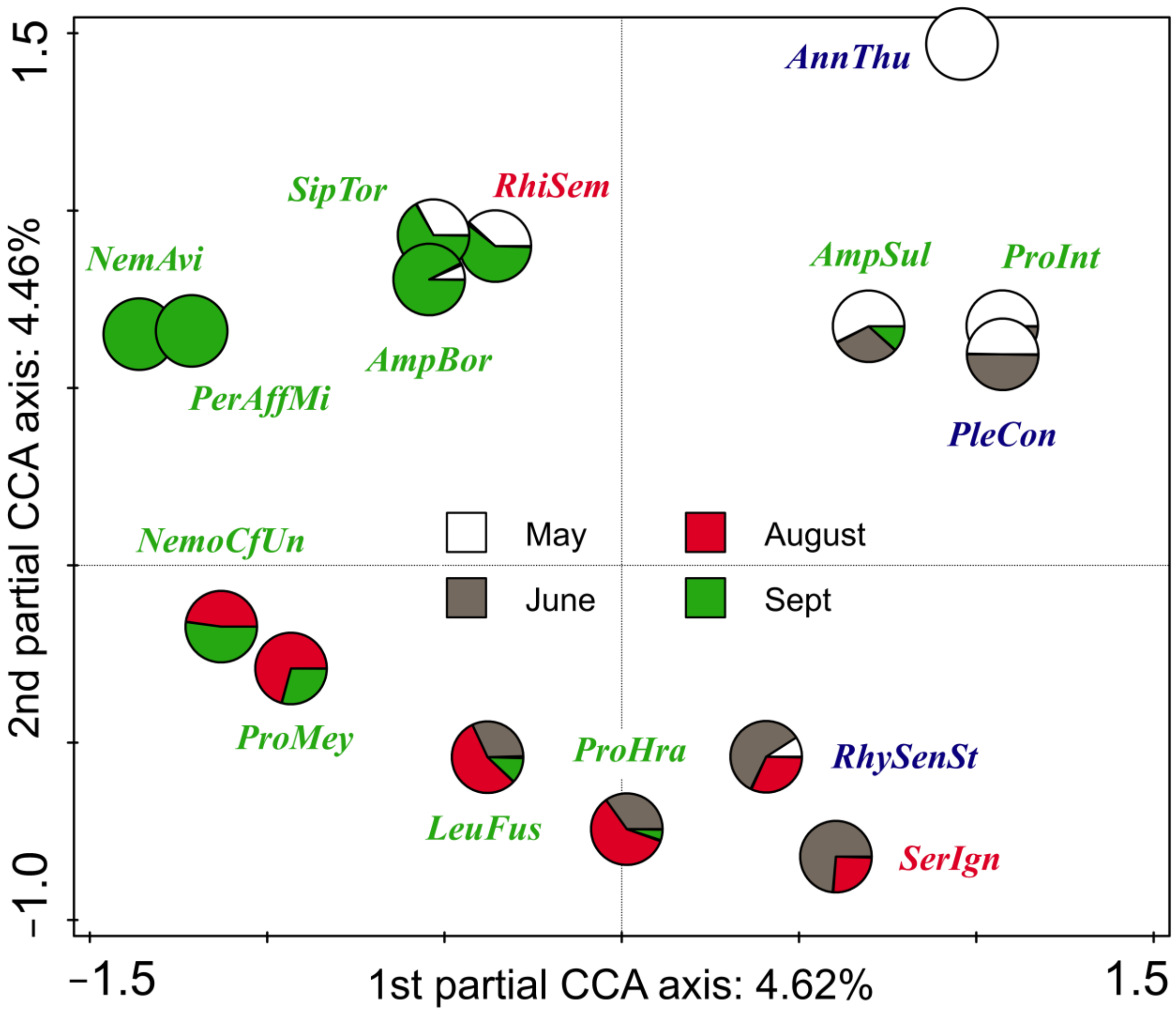

Figure S6. Species pie chart: the first 15 best fitting species are labelled (Ephemeroptera, Plecoptera and Trichoptera labaled in red, green and blue, respectively). Partial Canonical correspondence analysis (CCA) was used. Ordination axes were constrained by the factor 'Sampling time' which accounted for 11.62% of the explained variation. Test on first axis: pseudo-F = 0.7, p = 0.024; test on all axes: pseudo-F = 1.8, p = 0.001. Conditional effects (from the greatest to the lowest explained variation): 'September': explained variation = 4.1%, pseudo-F = 1.8, p (adj.) = 0.017; 'May': explained variation = 4.1%, pseudo-F = 1.9, p (adj.) = 0.017 and 'June': explained variation = 3.4%, pseudo-F = 1.6, p (adj.) = 0.02. Abbreviations: *AnnThu* = *Annitella thuringica*, *AmpBor* = *Amphinemura borealis*, *AmpSul* = *A. sulcicollis*, *LeuFus* = *Leuctra fusca*, *NemAvi* = *Nemoura avicularis*, *NemoCfUn* = *N.* cf. *uncinata*, *PerAffMic* = *Perlodes* aff. *microcephalus*, *PleCon* = *Plectrocnemia conspersa*, *ProHra* = *Protonemura hrabei*, *ProInt* = *P. intricata*, *ProMey* = *P. meyeri/nitida*, *RhiSem* = *Rhithrogena semicolorata*, *RhySenSt* = *Rhyacophila* sensu stricto (early instar larvae), *SerIgn* = *Serratella ignita*.

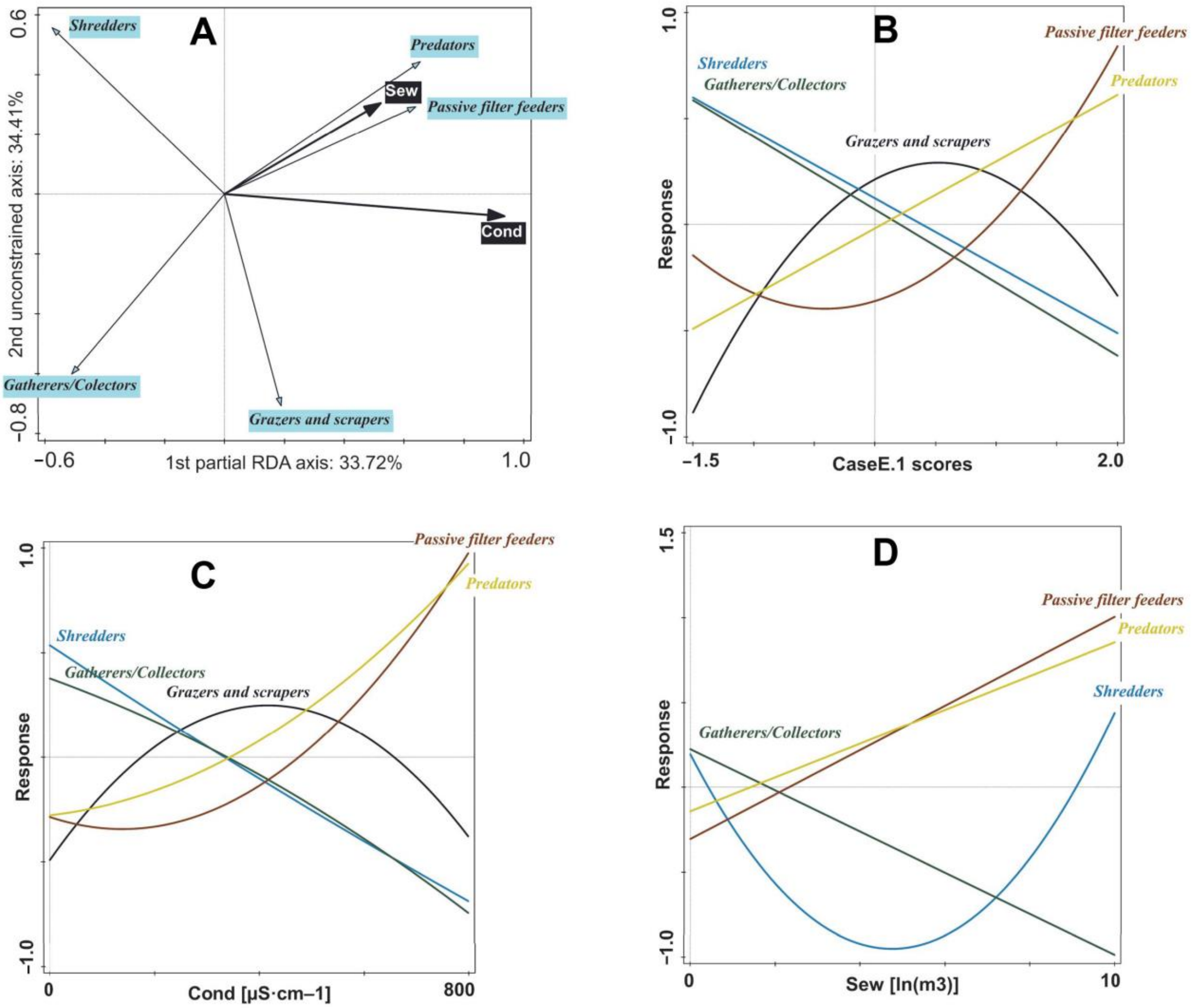

Figure S7. (**A**) Averaged feeding strategies + whole-plot environmental variables (EVs) biplot. (**B**) Plotted significant averaged feeding strategies response curves against 'CaseE.1 scores': fitted case scores of the '1st partial RDA axis' from (**A**). (**C**) Plotted significant averaged feeding strategies response curves against 'Conductivity (Cond)'. (**D**) Plotted significant averaged feeding strategies response curves against log-transformed 'Total volume of released sewage for three days prior to particular sampling time (Sew)'. (**A** and **B**) Partial Redundancy analysis (RDA) was used. The first ordination axis was constrained by the particular EVs selected by forward selection. 'Cond': explained variation = 32.5%, pseudo-F = 20.7, p (adj.) = 0.005 and 'Sew': explained variation = 6.4%, pseudo-F = 4.4, p (adj.) = 0.045. (**B**, **C** and **D**) Generalized linear models (GLMs) with gaussian distribution (with the *identity* link function) were used. Quadratic fit was chosen for the response where the fit was significantly improved compared to the linear one. See Table S8 for the statistics of relationship strengths.

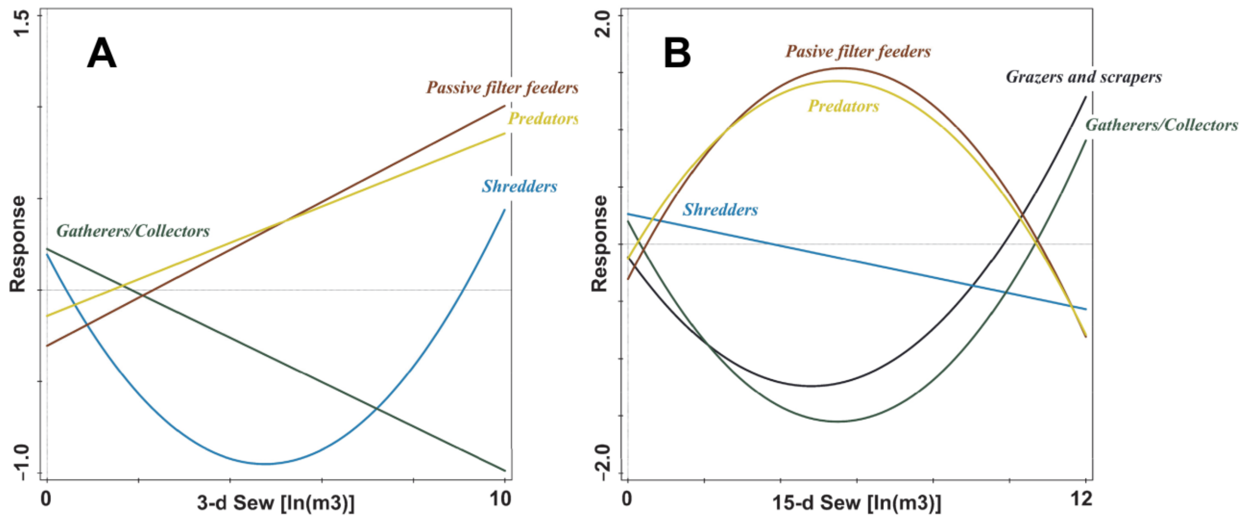

Figure S8. (**A**) Plotted significant averaged feeding strategies response curves against log-transformed 'Total volume of released sewage for three days prior to particular sampling Table 3. d Sew)' (**B**) Plotted significant averaged feeding stretegies response curves against log-transformed 'Total volume of released sewage for 15 days prior to particular sampling time (15-d Sew)'. (**A** and **B**) Generalised linear models (GLMs) with gaussian distribution (with the *identity* link function) were used. Quadratic fit was chosen for the response where the fit was thus improved. See Table S8 for the statistics of relationship strengths.

Table S8. The statistics of relationship strengths for plots in Figures S7 and S8. Abbreviations: Gat = Gatheres/Collectors, Gra = Grazers and scrapers, Pff = Passive filter feeders, Pre = Predators, Shr = Shredders.

	Feeding strategies				
	**Gat**	**Gra**	**Pff**	**Pre**	**Shr**
	$R^2 = 30.3\%$	$R^2 = 22.4\%$	$R^2 = 44.4\%$	$R^2 = 41.9\%$	$R^2 = 23.7\%$
**Fig. S7B**	$F_{1,46} = 20.0$	$F_{2,45} = 6.4$	$F_{2,45} = 17.9$	$F_{1,46} = 33.2$	$F_{2,45} = 14.3$
	$p < 0.001$	$p = 0.003$	$p < 0.001$	$p < 0.001$	$p < 0.001$
	$R^2 = 25.8\%$	$R^2 = 17.1\%$	$R^2 = 39.8\%$	$R^2 = 46.8\%$	$R^2 = 29.4\%$
**Fig. S7C**	$F_{1,46} = 16.0$	$F_{2,45} = 4.6$	$F_{2,45} = 14.9$	$F_{1,46} = 40.4$	$F_{2,45} = 19.1$
	$p < 0.001$	$p = 0.015$	$p < 0.001$	$p < 0.001$	$p < 0.001$
	$R^2 = 34.3\%$		$R^2 = 46.5\%$	$R^2 = 38.2\%$	$R^2 = 14.0\%$
**Figs. S7D and S8A**	$F_{1,46} = 24.0$	$F_{1,46} = 1.37$	$F_{1,46} = 40.0$	$F_{1,46} = 28.5$	$F_{2,45} = 3.7$
	$p < 0.001$	$p = 0.248$	$p < 0.001$	$p < 0.001$	$p < 0.034$
	$R^2 = 38.5\%$	$R^2 = 30.6\%$	$R^2 = 54.9\%$	$R^2 = 49.1\%$	$R^2 = 18.3\%$
**Fig. S8B**	$F_{2,45} = 14.1$	$F_{2,45} = 9.9$	$F_{2,45} = 27.4$	$F_{2,45} = 21.7$	$F_{1,46} = 10.3$
	$p < 0.001$	$p < 0.001$	$p < 0.001$	$p < 0.001$	$p = 0.002$

Table S9. Abundance and biomass of fish detected at the particulate sites during 20 May and 8 October in 2020. The occurrence of brown trout (*Salmo trutta*) in the Site 1 was observed during macrozoobenthos sampling ('X' represents the presence). Grey-filled cells represent detection of given fish category in given site. Abbreviations: Aux. site = Auxiliary site; NA = data not available.

Fish species	Abundance	May							October						
	Biomass	Site 1	Site 2	Site 2.2	Site 3	Aux. Site 3	Site 4.1	Site 4.2	Site 1	Site 2	Site 2.2	Site 3	Aux. Site 3	Site 4.1	Site 4.2
**Brown trout** (*Salmo trutta*) – wild	N	X	23	17	5	9	6	2	X	17	21	5	NA	4	0
	W [g]	NA	449	636	232	868	394	396	NA	347	1325	926	NA	1760	0
**Brown trout** (*Salmo trutta*) – fry	N	NA	0	0	0	3	0	0	NA	0	0	0	NA	0	0
	W [g]	NA	0	0	0	2	0	0	NA	0	0	0	NA	0	0
**Brown trout** (*Salmo trutta*) – stocked	N	NA	0	0	0	0	2	0	NA	0	0	0	NA	1	0
	W [g]	NA	0	0	0	0	494	0	NA	0	0	0	NA	490	0
**Rainbow trout** (*Oncorhynchus mykiss*) – stocked	N	NA	0	0	0	0	1	0	NA	0	0	0	NA	0	0
	W [g]	NA	0	0	0	0	397	0	NA	0	0	0	NA	0	0
**Eurasian ruffe** (*Gymnocephalus cernua*)	N	NA	1	0	0	0	0	0	NA	0	0	0	NA	0	0
	W [g]	NA	15	0	0	0	0	0	NA	0	0	0	NA	0	0
**European perch** (*Perca fluviatilis*)	N	NA	0	1	0	1	0	0	NA	0	0	2	NA	4	8
	W [g]	NA	0	8	0	24	0	0	NA	0	0	40	NA	176	462
**Common bream** (*Abramis brama*)	N	NA	0	0	0	0	0	0	NA	0	0	0	NA	5	4
	W [g]	NA	0	0	0	0	0	0	NA	0	0	0	NA	321	158
**Common minnow** (*Phoxinus phoxinus*)	N	NA	0	3	187	141	262	105	NA	0	17	36	NA	68	8
	W [g]	NA	0	17	539	694	1853	594	NA	0	99	202	NA	679	64
**Common minnow** (*Phoxinus phoxinus*) – fry	N	NA	0	0	0	0	0	23	NA	0	9	2	NA	1	0
	W [g]	NA	0	0	0	0	0	35	NA	0	7	1	NA	0.5	0
**Common roach (***Rutilus rutilus*)	N	NA	0	0	0	0	12	11	NA	0	1	0	NA	4	4
	W [g]	NA	0	0	0	0	483	453	NA	0	4	0	NA	193	264
**Common rud** (*Scardinius erythrophthalmus*)	N	NA	0	0	0	0	2	0	NA	0	0	0	NA	0	3
	W [g]	NA	0	0	0	0	50	0	NA	0	0	0	NA	0	55
**Chub** (*Squalius cephalus*)	N	NA	0	0	0	0	1	19	NA	0	0	0	NA	0	6
	W [g]	NA	0	0	0	0	212	3489	NA	0	0	0	NA	0	45
**Gudgeon** (*Gobio gobio*)	N	NA	0	0	0	0	0	9	NA	0	0	0	NA	0	9
	W [g]	NA	0	0	0	0	0	285	NA	0	0	0	NA	0	353
**Stone loach (***Barbatula barbatula*)	N	NA	1	8	0	0	0	3	NA	1	4	0	NA	0	1
	W [g]	NA	2	62	0	0	0	31	NA	10	48	0	NA	0	12
**Brook lamprey** (*Lampetra planeri*) – adult	N	NA	0	0	0	0	0	0	NA	1	1	0	NA	0	0
	W [g]	NA							NA	7	8	0	NA	0	0
**Brook lamprey** (*Lampetra planeri*) – larvae	N	NA	9	10	0	0	0	0	NA	3	2	0	NA	0	0
	W [g]	NA	60	52	0	0	0	0	NA	8	10	0	NA	0	0
**TOTAL**	N	**NA**	**34**	**39**	**192**	**154**	**286**	**172**	**NA**	**22**	**55**	**45**	**NA**	**87**	**43**
	W [g]	**NA**	**536**	**775**	**771**	**1588**	**3883**	**5283**	**NA**	**372**	**1501**	**1169**	**NA**	**3619**	**1413**

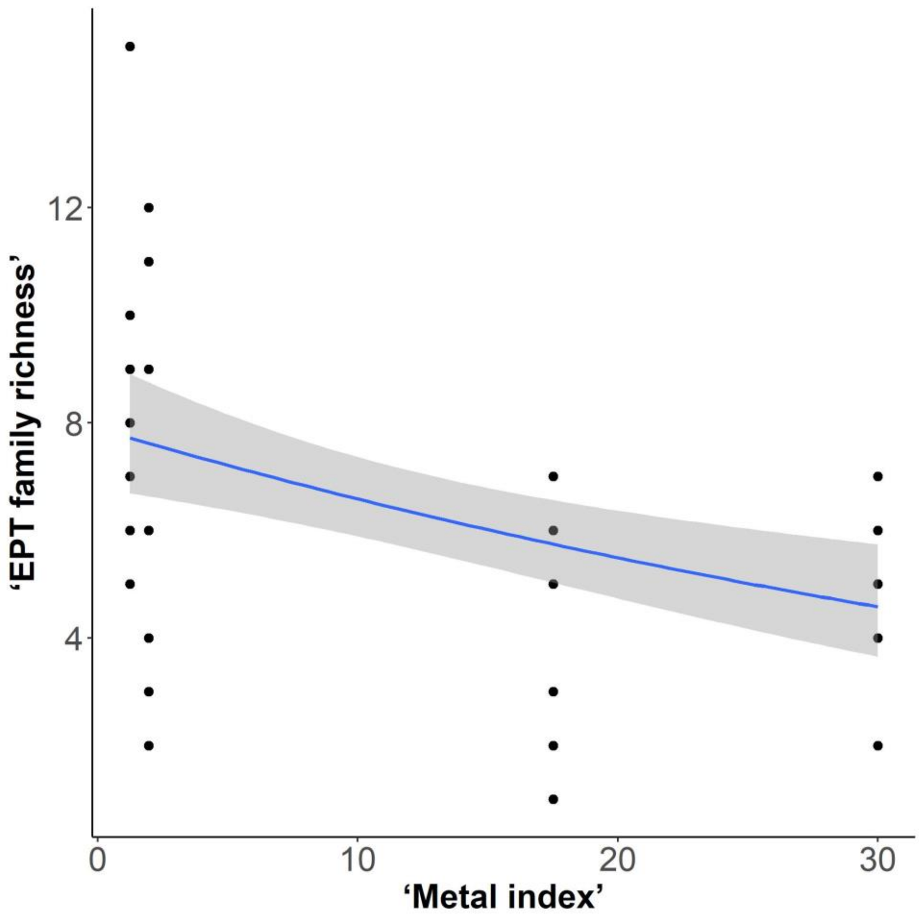

Figure S9. Plotted significant relationship between EPT family richness and Metal index (χ^2_1 = 13.42, $p <$ 0.001). A generalized linear model with Poisson distribution was used. The grey area represents 95% confidence region.

References

1. Grabic, R.; Fick, J.; Lindberg, R.H.; Fedorova, G.; Tysklind, M. Multi-residue method for trace level determination of pharmaceuticals in environmental samples using liquid chromatography coupled to triple quadrupole mass spectrometry. *Talanta* **2012**, *100*, 183–195, doi:10.1016/j.talanta.2012.08.032.
2. Grabicova, K.; Grabic, R.; Fedorova, G.; Kolarova, J.; Turek, J.; Brooks, B.W.; Randak, T. Psychoactive pharmaceuticals in aquatic systems: A comparative assessment of environmental monitoring approaches for water and fish. *Environmental Pollution* **2020**, *261*, 114150, doi:10.1016/j.envpol.2020.114150.
3. Lindberg, R.H.; Ostman, M.; Olofsson, U.; Grabic, R.; Fick, J. Occurrence and behaviour of 105 active pharmaceutical ingredients in sewage waters of a municipal sewer collection system. *Water Research* **2014**, *58*, 221–229, doi:10.1016/j.watres.2014.03.076.
4. Giddings, E. M.; Hornberger, M. I.; Hadley, H. K. Trace-metal concentrations in sediment and water and health of aquatic macroinvertebrate communities of streams near Park City, Summit County, Utah; In Utah; Science for a Changing World: Salt Lake City, Utah, 2001; pp. 9–10, doi:10.3133/wri014213.
